# Diaphragm Thickness and Contraction During Non-Invasive Ventilation: An Ultrasound Study

**DOI:** 10.3390/children12040470

**Published:** 2025-04-06

**Authors:** Stefano Nobile, Annamaria Sbordone, Nicola Salce, Giovanni Scognamiglio, Alessandro Perri, Simona Fattore, Giorgia Prontera, Lucia Giordano, Milena Tana, Giovanni Vento

**Affiliations:** 1 Neonatal Unit, Fondazione Policlinico Universitario “A. Gemelli”, 00168 Rome, Italy; 2 Neonatal Unit, Policlinico Casilino, 00169 Rome, Italy; 3 Cardiovascular Unit, Policlinico San Martino, 16132 Genova, Italy

**Keywords:** infant, non-invasive ventilation, nasal continuous airway pressure, lung ultrasound, work of breathing

## Abstract

Objectives: Non-invasive ventilation (NIV) is a widely used treatment for neonatal respiratory distress syndrome (RDS). Data on diaphragm contractility and thickness during NIV is scarce. We aimed to describe changes in diaphragm thickness/contractility during NIV and to explore associations with NIV discontinuation failure. Methods: This is a single-center prospective study. Diaphragmatic ultrasound was performed weekly during NIV, then within 7 days from NIV discontinuation. Diaphragm thickness was measured at end-inspiration (DTI) and end-expiration (DTE). Diaphragm thickening fraction (DTF) was calculated as (DTI-DTE/DTE). The clinical characteristics of patients and NIV discontinuation failure were recorded. Univariate analysis, logistic regression and linear regression were performed to describe diaphragm features during NIV and associations with NIV discontinuation failure. Results: We studied 17 NIV cycles (median duration 21 days). Median DTE increased from 0.12 cm (SD 0.05) at the start of NIV to 0.15 cm (SD 0.04) at NIV discontinuation. The mean DTF decreased from 32.8 (SD 16.8) at the start of NIV to 25.6 (SD 8.9) at NIV discontinuation. NIV discontinuation failure occurred in 23.5% of infants and was associated with higher DTI and DTE at the start of NIV and with a more pronounced decrease in DTI and DTE over the NIV cycle, compared to infants with NIV discontinuation success. There were no differences in neonatal outcomes between the infants with NIV discontinuation failure vs. success. We did not find any significant predictors of NIV failure. Conclusions: Diaphragm thickness increased, whereas DTF decreased over time on NIV in preterm infants with RDS. NIV duration was not associated with changes in diaphragm trophism. NIV discontinuation failure was associated with thicker diaphragm at the start of NIV, as well as with a reduction in diaphragm trophism over the NIV cycle.

## 1. Introduction

Non-invasive ventilation (NIV) refers to providing respiratory support without the use of an intratracheal airway, in contrast to invasive mechanical ventilation (MV), which typically involves the presence of an endotracheal tube. The primary physiological goals of NIV are to improve gas exchange and reduce the work of breathing by reducing the load on the respiratory muscles [1]. NIV can be used either as a primary therapy (to avoid invasive ventilation) or as a secondary therapy (after weaning from invasive ventilation) in newborns with respiratory distress syndrome (RDS). RDS more often affects preterm infants than full-term infants, with preterm infants typically requiring NIV for longer periods [1]. Breathing is primarily carried out by the respiratory muscles, with the diaphragm being the key muscle for inspiration. Various clinical and mechanical factors can impair the function of these respiratory muscles, potentially leading to respiratory muscle failure. In newborns, the diaphragm serves as the primary inspiratory muscle, and there are notable structural and functional differences compared to children and adults. These differences include a highly compliant rib cage, the horizontal alignment of the ribs (which limits the action of accessory respiratory muscles), a smaller zone of apposition for the diaphragm, and a reduced functional reserve due to reduced expression of fatigue-resistant muscle fibers [2].

In addition to clinical assessment, lung ultrasound (LUS) is widely used to assess respiratory distress syndrome and other respiratory issues in newborns [3,4,5]. In particular, NIV weaning is currently guided by clinical assessment, and individual judgement, rather than objective factors, usually guides this process. Few studies reporting on diaphragm ultrasound assessment have been conducted in infants on NIV to predict its discontinuation. In particular, Nour et al. assessed the accuracy of LUS in predicting successful weaning from nasal continuous positive airway pressure (nCPAP) and found that diaphragm features were not useful in predicting weaning, in contrast with the LUS score [6]. However, the authors performed ultrasound only twice, just before weaning and three hours after nCPAP discontinuation. The most used indicators of diaphragm performance are diaphragm thickness at end-inspiration (DTI), diaphragm thickness at end-expiration (DTE), and diaphragm thickening fraction [DTF = (DTI-DTE)/DTE] [6]. We hypothesize that performing LUS more frequently during NIV could enhance the understanding of diaphragm features and ultimately help predict NIV discontinuation more precisely. Our aim was to describe changes in diaphragm thickness/contractility in relation to the entire duration of nasal ventilatory support and to explore associations with NIV discontinuation failure.

## 2. Materials and Methods

This was a prospective observational study conducted at an advanced Neonatal Intensive Care Unit (NICU) at Fondazione Policlinico Gemelli IRCCS in Rome (Italy) between February 2021 and February 2022. We studied preterm infants born < 37 weeks of gestation, who required NIV as primary therapy for RDS or after extubation during their NICU admission. Infants requiring NIV for >2 weeks were included in a secondary analysis. Infants with chromosomal abnormalities, major malformations, gestational age < 23 weeks, and need for palliative care were excluded. Clinical characteristics of patients, including gestational and postnatal age, sex, length, weight, comorbidities, nutritional and other data, were recorded.

NIV strategies for the treatment of neonatal RDS included nCPAP and nasal intermittent mandatory ventilation, which were administered by VN500 ventilators (Draeger, Lubeck, Germany). In our unit, NIV is the initial strategy for neonatal RDS, with the exception of asphyxiated newborns, who require intubation in the delivery room, and NIV failure criteria, which follow specific unit guidelines. Appropriately sized nasal prongs and masks were alternated at nurses’ discretion in order to maximize NIV efficacy and reduce the risk of complications (e.g., skin lesions, gastric distention) according to local protocols. NIV pressures were gradually weaned based on the infant’s tolerance, according to local protocols, and available evidence: FiO_2_ ≤ 0.25, respiratory rate ≤ 60/minute, Silverman score < 3, no significant apnea/bradycardia [7]. The oxygen saturation targets were 90–95% for preterm infants born < 37 weeks of gestation; the pCO_2_ targets were 45–55 mmHg. All infants on NIV received caffeine therapy as per the unit guidelines, throughout the NIV cycle.

NIV failure was defined as the requirement for its restart within 7 days from discontinuation if any of the following occurred: increased work of breathing (respiratory rate > 75 per minute, Silverman score > 3), significant apnea/bradycardia, FiO_2_ > 0.30, respiratory acidosis (pCO_2_ > 65 mmHg and pH < 7.20). This time frame was arbitrarily chosen based on our clinical experience.

Diaphragmatic ultrasound was performed weekly for the entire duration of non-invasive ventilatory support, then at least once during spontaneous breathing, within 7 days from the NIV discontinuation. LUS was performed by two skilled operators (SN and NS), and the LUS score was calculated as previously reported [8]. A 12 MHz linear probe with a resolution limit of 0.01 mm was used. The infants were kept supine during examination, and the probe was longitudinally applied on the right mid-axillary line (diaphragm apposition zone). In a previous publication, the agreement between the two operators was high [9].

**Figure 1:**
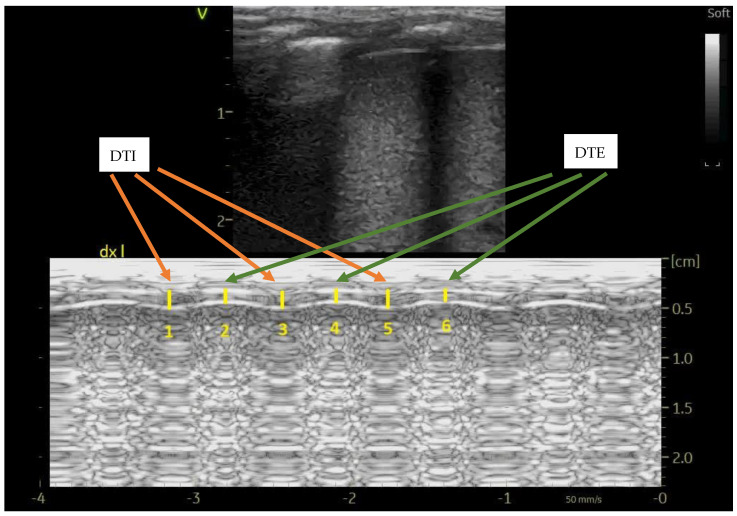
Diaphragm ultrasound in M-mode showing the DTI (average value of measures number 1, 3 and 5) and DTE measurements (average value of measures number 2, 4 and 6). The probe was longitudinally applied on the right mid-axillary line (diaphragm apposition zone).

Diaphragm thickness at end-inspiration (DTI) and end-expiration (DTE) were recorded, and diaphragm thickening fraction was calculated as (DTI-DTE)/DTE; at least three measures in M-mode were averaged, as depicted in Figure 1; diaphragm atrophy during mechanical ventilation was defined as ≥ 10% decrease in DTE [9].

We studied the association between (1) the duration of nasal ventilatory support and changes in diaphragm thickness/contractility (calculated weekly), as well as (2) the association between NIV discontinuation failure and changes in thickness and the thickening fraction of the diaphragm.

The results are reported as percentages, mean (standard deviation, SD) for normally distributed variables and as median (interquartile range, IQR) for non-normally distributed variables. Univariate analysis (the *t*-test, the Mann–Whitney test, and the chi-squared test, as appropriate) were used to explore associations. We used logistic regression to assess the relationship between patient characteristics and NIV discontinuation failure. Risk factors for NIV discontinuation failure with a *p* value < 0.20 on the univariate analyses were entered into multivariate logistic regression, and the best model was chosen based on the Hosmer–Lemeshow and the Nagelkerke tests. Linear regression was performed to evaluate the association between duration of nasal ventilatory support and change in diaphragm measures. SPSS (version 25.0, IBM, Armonk, NY, USA) was used to compute statistics. At the time of the research conceptualization, there were no studies with the same objectives; thus, we designed this as a pilot study, without a formal sample size calculation. This study was approved by the local Ethics Committee (protocol number 0050044/20), and written informed consent was obtained from the caregivers of each infant.

## 3. Results

Seventeen patients were enrolled, and 17 NIV cycles were assessed during the study period (11 NIV cycles lasted >2 weeks). Demographics and clinical characteristics of the patients are shown in Table 1. NIV was the primary mode of assistance for five infants, whereas it was preceded by tracheal ventilation in 12 infants.

The median duration of NIV cycles was 21 days (IQR 6–34); the median day of life at the start of IMV was 7 days (IQR 1–9). The mean positive end-expiratory pressure (PEEP) at the start of NIV was 7 cmH_2_0 (SD 1.6); The mean LUS score at the start of NIV was 8 (SD 3.6).

The mean DTE on the first NIV day was 0.12 cm (SD 0.05); the mean DTE on the last NIV day was 0.15 cm (SD 0.04).

The mean DTI on the first NIV day was 0.16 cm (SD 0.06); the mean DTI on the last NIV day was 0.19 cm (SD 0.05).

The mean DTF on the first NIV day was 32.8 (SD 16.8); the mean DTF on the last NIV day was 25.6 (SD 8.9).

### 3.1. NIV Discontinuation Failure

Four patients (23.5%) experienced NIV failure within 7 days from discontinuation. There were no significant differences in clinical characteristics between the infants with NIV discontinuation failure compared to those with NIV discontinuation success, except for DTI and DTE at the start of NIV and the changes in DTI and DTE between the end and the start of the NIV cycle (Table 2).

There were no significant differences regarding outcomes between the infants with NIV discontinuation failure and those with NIV discontinuation success: hsPDA (0 vs. 4, *p* = 0.519), hsPDA during NIV (0 vs. 2, *p* = 1.000), BPD (1 vs. 2, *p* = 1.000), length of stay [104 (38) vs. 63 (48), *p* = 0.124], severe IVH (0 vs. 2, *p* = 0.771), severe ROP (0 vs. 1, *p* = 0.210), NEC (0 vs. 1, *p* = 1.000), LOS (3 vs. 4, *p* = 0.593).

The logistic regression analysis could not find any significant predictors of NIV failure. The tested variables were gestational age, previous MV, DTI/DTE change between the start and end of NIV (Nagelkerke R^2^ 0.300, Hosmer Lemeshow 0.900).

A linear regression analysis was performed to assess factors associated with changes in DTE; the included variables were NIV duration, gestational age, sepsis, and NPO duration. The R^2^ of the model was 0.452. There were no significant associations between these variables and DTE change: NIV duration B = 0.001, 95%CI −0.002–0.004, *p* = 0.356; GA B = −0.001, 95%CI −0.004–0.002, *p* = 0.507; sepsis B = 0.007, 95% CI −0.073–0.087, *p* = 0.859; NPO days B = 0.001, 95% CI −0.004–0.006, *p* = 0.578.

### 3.2. Infants with NIV Duration >2 Weeks

Eleven infants had prolonged NIV cycles, lasting more than 2 weeks. As expected, they were smaller and less mature than the whole population. The patients’ characteristics are shown in Table 3.

All infants had previous MV, with a mean duration of 3.2 days (SD 2.8); none of them needed reintubation after MV discontinuation. The mean NIV duration was 35.3 days (SD 10.8); 4/11 infants (36.4%) had NIV discontinuation failure. The mean age at the start of IMV was 12.8 days (SD 8.9). The mean PEEP at the start of NIV was 7.6 cmH_2_0 (SD 1.6); the mean LUS score at the start of NIV was 8 (SD 3.6).

The mean number of diaphragm ultrasound measurements was 5.3 (SD 2.7) per patient.

The mean DTE on the first NIV day was 0.10 cm (SD 0.03); the mean DTE on the last NIV day was 0.15 cm (SD 0.05).

The mean DTI on the first NIV day was 0.13 cm (SD 0.04); the mean DTI on the last NIV day was 0.19 cm (SD 0.06).

The mean DTF on the first NIV day was 34.7 (SD 18.7); the mean DTF on the last NIV day was 23.1 (SD 8.4).

### 3.3. NIV Discontinuation Failure After Prolonged (>2 Weeks) NIV Courses

Four patients (36.4%) experienced NIV failure within 7 days from discontinuation. There were no significant differences in clinical characteristics between infants with NIV discontinuation failure compared to those with NIV discontinuation success (Table 4).

There were no significant differences regarding outcomes between infants with NIV discontinuation failure compared to those with NIV discontinuation success: hsPDA (0 vs. 4, *p* = 0.194), hsPDA during NIV (0 vs. 2, *p* = 0.491), BPD (1 vs. 2, *p* = 1.000), length of stay [104 (38) vs. 99 (37), *p* = 0.821], severe IVH (0 vs. 2, *p* = 0.462), severe ROP (0 vs. 1, *p* = 0.402), NEC (0 vs. 1, *p* = 1.000), LOS (2 vs. 6, *p* = 0.491).

A logistic regression analysis could not find any significant predictors of NIV failure. The tested variables were gestational age, DTE change between the start and the end of NIV, and NPO days (Nagelkerke R^2^ 0.134, Hosmer Lemeshow 0.420).

A linear regression analysis was performed to assess factors associated with changes in DTE; the included variables were NIV duration, gestational age, sepsis, and NPO duration. The R^2^ of the model was 0.156. There were no significant associations between these variables and DTE change: NIV duration B = 0.002, 95% CI −0.004–0.007, *p* = 0.479; GA B = −0.001, 95%CI −0.005–0.003, *p* = 0.686; sepsis B = 0.010, 95% CI −0.102–0.122, *p* = 0.828; NPO days B = 0.002, 95% CI −0.006–0.009, *p* = 0.634.

## 4. Discussion

We reported on the changes in diaphragm thickness and contractility during NIV administered to preterm infants with RDS. We found that DTI and DTE increased over time, even if we did not find associations between the duration of NIV course and diaphragm thickness. Moreover, the DTF decreased over time, suggesting a decreased respiratory effort, probably favored by NIV, in the context of a growing diaphragm. Alternative explanations of the DTF decrease might be a reduced diaphragmatic effort due to RDS resolution, the occurrence of diaphragm fatigue/functional unloading or even structural and/or functional changes in contractile units of muscle fibers.

NIV duration was not significantly associated with changes in diaphragm muscle growth (trophism, e.g., DTE). NIV can influence respiratory mechanics, and has been associated with several advantages, including reduced work of breathing, rib cage and airway stabilization, achievement and maintenance of functional residual capacity, alveolar recruitment resulting in improved ventilation-perfusion matching and better gas exchange [10,11]. We speculate that our data reflect a reduced diaphragm load in the context of a resolving RDS in preterm infants, in line with these physiologic premises. It is important to note that most of the study infants had previously received invasive ventilation, which may have influenced the observed results.

NIV discontinuation failure was experienced by 23.5% of infants and was associated (only at univariate analysis) with higher DTI and DTE at NIV start, and with a more pronounced decrease in DTI and DTE over the NIV cycle compared to the infants with NIV discontinuation success. In other words, infants with NIV discontinuation failure, compared to infants who could be successfully weaned, had a thicker diaphragm at the start of NIV, but experienced a higher degree of thickness reduction all over the NIV cycle. Identifying factors associated with decreased diaphragm trophism could be useful in preventing NIV discontinuation failure. We could not find any other significant differences between the two groups and hypothesize that other factors, such as nutritional or inflammatory issues could have influenced diaphragm trophism, as previously suggested [12,13]. In a previous study, Nour et al. assessed the role of lung and diaphragm ultrasound in the prediction of successful weaning from nCPAP in preterm infants [6]. They performed ultrasound scans twice, just before and 3 h after weaning off nCPAP, and found that a LUS score < 6–7 was significantly associated with successful discontinuation, whereas diaphragmatic excursion and DTF were not significant predictors. The authors reported that the DTF was significantly lower in the successful weaning group compared to the failure group at univariate analysis, but this difference was not statistically significant at multivariate analysis. Other studies reported similar findings regarding diaphragm excursion and DTF, which were, respectively, lower and higher in infants with weaning failure compared to weaning success [14]. We observed a lower DTF in the successful NIV weaning group compared to the failure group, even if the difference was not statistically significant. In our opinion, also considering the small sample size of our study, this observation might reflect a higher diaphragmatic effort experienced by infants who eventually failed NIV discontinuation. Different clinical characteristics and practices, as well as the different timing of ultrasound assessment in these studies, may explain the observed variability in the results. In future studies, the possibility of enhancing diaphragm function may be explored with proper interventions, e.g., physiotherapy or other ventilation techniques, such as non-invasive neurally-adjusted ventilatory assist (NIV-NAVA). Besides diaphragm ultrasound, methods such as transcutaneous electromyography may be helpful in assessing diaphragm function, as previously shown [15].

We also found that infants with prolonged NIV cycles were smaller and less mature at birth compared to those with shorter cycles; all of them had undergone previous MV for RDS. In this subgroup, DTI and DTE increased over the NIV course, whereas DTF showed a decrease, similar to the trend in the whole population. A higher proportion of infants with prolonged NIV experienced NIV discontinuation failure compared to the whole population.

This study has some limitations. First, the small sample size and the lack of a control group limit firm conclusions. Secondly, our data may not be applicable to other populations, and local practices could be different compared to other centers (including the timing of LUS evaluations). For instance, most of our infants had received invasive ventilation prior to NIV, and caffeine was started by day 1 of life, as per institutional guidelines in every preterm infant with RDS. Furthermore, we studied very thin diaphragms, and the margin of error could therefore be high; however, as in other studies conducted among infants, the agreement between operators was good, and the measures of at least two respiratory cycles were averaged to minimize the risk of errors.

In conclusion, this prospective observational study in preterm infants showed that diaphragm thickness increased, whereas DTF decreased over time on NIV, indicating muscle growth and reduced muscle effort. NIV duration was not significantly associated with changes in diaphragm trophism. NIV discontinuation failure was associated with thicker diaphragm at the start of NIV, as well as with a reduction in diaphragm trophism over the NIV cycle. NIV discontinuation failure was not associated with clinical outcomes. Further studies are needed to confirm these findings: larger samples and collection of other relevant data, including nutritional and pharmacological details, are warranted in order to optimize and personalize non-invasive ventilatory assistance in preterm infants. Moreover, future studies should assess the effectiveness of proper interventions to enhance diaphragm function and reduce the need for ventilatory assistance in preterm infants.

## Figures and Tables

**Table 1:** Demographics and clinical characteristics of the study population.

Gestational age at birth, weeks	28^6/7^ (27^3/7^–32^1/7^)
Birth weight, g	1178 (820–1670)
Male sex, *n* (%)	11 (64.7)
Small for gestational age (SGA), *n* (%)	2 (11.8)
Singletons, *n* (%)	9 (52.9)
Clinical chorioamnionitis, *n* (%)	2 (11.8)
Sepsis, *n* (%)	5 (29.4)
Surfactant doses, median (IQR)	1 (0–2)
Hemodynamically significant patent ductus arteriosus (hsPDA), *n* (%)	4 (23.5)
hsPDA during NIV, *n* (%)	2 (11.8)
Previous mechanical ventilation (MV), *n* (%)	12 (70.6)
Previous diaphragm atrophy during MV, *n* (%)	4/11 (36.4)
Birth asphyxia, *n* (%)	2 (11.8)
Bronchopulmonary dysplasia (BPD), *n* (%)	3 (17.6)
Necrotizing enterocolitis (NEC) grade 3–4, *n* (%)	1 (5.9)
Intraventricular hemorrhage (IVH) grade ≥3, *n* (%)	2 (11.8)
Retinopathy of prematurity (ROP) stage ≥3, *n* (%)	1 (5.9)
Admission (days), median (IQR)	73 (23–99)

**Table 2:** Clinical characteristics of patients with NIV discontinuation failure vs. success.

	NIV Discontinuation Failure, *n*. 4	NIV Discontinuation Success, *n*. 13	*p*-Value
Female gender, *n* (%)	2 (50.0)	2 (15.4)	0.538
Birth weight, grams; median (IQR)	999 (618–1330)	1420 (803–1890)	0.296
Gestational age, days; median (IQR)	194 (189–200)	219 (192–226)	0.412
SGA, *n* (%)	1 (25.0)	1 (7.7)	0. 426
Previous MV, *n* (%)	4 (100.0)	8 (61.5)	0.261
Previous DA during MV, *n* (%)	2 (50.0)	2 (25.0)	1.000
Diaphragm atrophy, *n* (%)	1 (25.0)	5 (38.5)	1.000
NIV duration, days; median (IQR)	37 (24–41)	15 (5–34)	0.130
Age at the start of NIV, days; median (IQR)	8 (6–23)	4 (1–13)	0.245
Nihil per os (NPO) days; mean (SD)	5 (2)	5 (9)	0.824
PEEP at the start of NIV, cmH_2_0; median (IQR)	8 (2)	7 (1)	0.212
DTI at the start of NIV, cm; mean (SD)	0.22 (0.05)	0.13 (0.04)	0.001
DTE at the start of NIV, cm; mean (SD)	0.17 (0.04)	0.09 (0.03)	<0.001
DTF at the start of NIV, %; mean (SD)	24.8 (9.5)	37.2 (18.7)	0.151
Last DTI, cm; mean (SD)	0.18 (0.03)	0.19 (0.06)	0.697
Last DTE, cm; mean (SD)	0.14 (0.02)	0.16 (0.05)	0.511
Last DTF, %; mean (SD)	28.7 (9.4)	23.9 (8.7)	0.305
DTI change between the start and discontinuation of NIV, cm; mean (SD)	−0.04 (0.04)	0.07 (0.04)	<0.001
DTE change between the start and discontinuation of NIV, cm; mean (SD)	−0.03 (0.02)	0.06 (0.05)	<0.001
DTF change between the start and discontinuation of NIV, %; median (IQR)	−12.9 (−40.0–2.4)	2.4 (−16.4–7.6)	0.350

**Table 3:** Characteristics of infants with prolonged NIV cycles.

Gestational age at birth, weeks; mean (SD)	28^0/7^ (0.3)
Birth weight, g; mean (SD)	886 (299)
Male sex, *n* (%)	6 (54.5)
SGA, *n* (%)	2 (18.2)
Singletons, *n* (%)	5 (45.5)
Clinical chorioamnionitis, *n* (%)	2 (18.2)
Outborn, *n* (%)	1 (9.1)
Birth asphyxia, *n* (%)	1 (9.1)
Sepsis, *n* (%)	5 (45.5)
Surfactant doses, mean (SD)	1.6 (0.8)
hsPDA, *n* (%)	4 (36.4)
hsPDA during NIV, *n* (%)	2 (18.2)
BPD, *n* (%)	3 (27.3)
NEC grade 3–4, *n* (%)	1 (9.1)
IVH grade ≥3, *n* (%)	2 (18.2)
ROP stage ≥3, *n* (%)	1 (9.1)
Admission (days), mean (SD)	101 (35)

**Table 4:** Clinical characteristics of infants with NIV discontinuation failure vs. success (NIV >2 weeks).

	NIV Discontinuation Failure, *n*. 4	NIV Discontinuation Success, *n*. 7	*p*-Value
Female gender, *n* (%)	2 (50.0)	3 (42.9)	1.000
Birth weight, grams; median (IQR)	982 (370)	893 (294)	0.670
Gestational age, days; median (IQR)	194 (6.1)	195 (15.3)	0.905
SGA, *n* (%)	1 (25.0)	1 (14.3)	1.000
Diaphragm atrophy, *n* (%)	1 (25.0)	0 (0)	0.364
NIV duration, days; mean (SD)	34 (9)	33 (14)	0.954
Age at NIV start, days; mean (SD)	12 (10)	13 (9)	0.832
NPO days; mean (SD)	5 (2)	9 (10)	0.291
PEEP at NIV start, cmH_2_0; median (IQR)	8.0 (2.2)	7.3 (1.4)	0.562
DTI at NIV start, cm; mean (SD)	0.16 (0.05)	0.11 (0.03)	0.082
DTE at NIV start, cm; mean (SD)	0.11 (0.04)	0.09 (0.02)	0.160
DTF at NIV start, %; mean (SD)	40.7 (17.2)	31.3 (19.9)	0.450
Last DTI, cm; mean (SD)	0.22 (0.08)	0.17 (0.04)	0.311
Last DTE, cm; mean (SD)	0.18 (0.07)	0.14 (0.04)	0.365
Last DTF, %; mean (SD)	24.2 (8.5)	22.6 (9.0)	0.783
DTI change between NIV discontinuation and NIV start, cm; mean (SD)	0.06 (0.07)	0.06 (0.04)	0.904
DTE change between NIV discontinuation and NIV start, cm; mean (SD)	0.06 (0.07)	0.05 (0.04)	0.758
DTF change between NIV discontinuation and NIV start, %; median (IQR)	−16.5 (22.0)	−8.7 (21.4)	0.577

## Data Availability

The raw data supporting the conclusions of this article will be made available by the authors on request.

## Abbreviations

The following abbreviations are used in this manuscript:

NIV	Non-invasive ventilation
RDS	Respiratory distress syndrome
DTI	Diaphragm thickness at end-inspiration
DTE	Diaphragm thickness at end-expiration
LUS	Lung ultrasound
nCPAP	Nasal continuous positive airway pressure
DTF	Diaphragm thickening fraction
SD	Standard deviation
IQR	Interquartile range
SGA	Small for gestational age
hsPDA	Haemodynamically significant patent ductus arteriosus
MV	Mechanical ventilation
BPD	Bronchopulmonary dysplasia
NEC	Necrotising enterocolitis
IVH	Intraventricular haemorrhage
ROP	Retinopathy of prematurity
PEEP	Positive end-expiratory pressure
NPO	Nihil per os
